# MASK 2017: ARIA digitally-enabled, integrated, person-centred care for rhinitis and asthma multimorbidity using real-world-evidence

**DOI:** 10.1186/s13601-018-0227-6

**Published:** 2018-10-25

**Authors:** J. Bousquet, S. Arnavielhe, A. Bedbrook, M. Bewick, D. Laune, E. Mathieu-Dupas, R. Murray, G. L. Onorato, J. L. Pépin, R. Picard, F. Portejoie, E. Costa, J. Fonseca, O. Lourenço, M. Morais-Almeida, A. Todo-Bom, A. A. Cruz, J. da Silva, F. S. Serpa, M. Illario, E. Menditto, L. Cecchi, R. Monti, L. Napoli, M. T. Ventura, G. De Feo, D. Larenas-Linnemann, M. Fuentes Perez, Y. R. Huerta Villabolos, D. Rivero-Yeverino, E. Rodriguez-Zagal, F. Amat, I. Annesi-Maesano, I. Bosse, P. Demoly, P. Devillier, J. F. Fontaine, J. Just, T. P. Kuna, B. Samolinski, A. Valiulis, R. Emuzyte, V. Kvedariene, D. Ryan, A. Sheikh, P. Schmidt-Grendelmeier, L. Klimek, O. Pfaar, K. C. Bergmann, R. Mösges, T. Zuberbier, R. E. Roller-Wirnsberger, P. Tomazic, W. J. Fokkens, N. H. Chavannes, S. Reitsma, J. M. Anto, V. Cardona, T. Dedeu, J. Mullol, T. Haahtela, J. Salimäki, S. Toppila-Salmi, E. Valovirta, B. Gemicioğlu, A. Yorgancioglu, N. Papadopoulos, E. P. Prokopakis, S. Bosnic-Anticevich, R. O’Hehir, J. C. Ivancevich, H. Neffen, E. Zernotti, I. Kull, E. Melen, M. Wickman, C. Bachert, P. Hellings, S. Palkonen, C. Bindslev-Jensen, E. Eller, S. Waserman, M. Sova, G. De Vries, M. van Eerd, I. Agache, T. Casale, M. Dykewickz, R. N. Naclerio, Y. Okamoto, D. V. Wallace, MASK study group, J. Bousquet, P. W. Hellings, W. Aberer, I. Agache, C. A. Akdis, M. Akdis, M. R. Alberti, R. Almeida, F. Amat, R. Angles, I. Annesi-Maesano, I. J. Ansotegui, J. M. Anto, S. Arnavielle, E. Asayag, A. Asarnoj, H. Arshad, F. Avolio, E. Bacci, C. Bachert, I. Baiardini, C. Barbara, M. Barbagallo, I. Baroni, B. A. Barreto, X. Basagana, E. D. Bateman, M. Bedolla-Barajas, A. Bedbrook, M. Bewick, B. Beghé, E. H. Bel, K. C. Bergmann, K. S. Bennoor, M. Benson, L. Bertorello, A. Z. Bialoszewski, T. Bieber, S. Bialek, C. Bindslev-Jensen, L. Bjermer, H. Blain, F. Blasi, A. Blua, M. Bochenska Marciniak, I. Bogus-Buczynska, A. L. Boner, M. Bonini, S. Bonini, C. S. Bosnic-Anticevich, I. Bosse, J. Bouchard, L. P. Boulet, R. Bourret, P. J. Bousquet, F. Braido, V. Briedis, C. E. Brightling, J. Brozek, C. Bucca, R. Buhl, R. Buonaiuto, C. Panaitescu, M. T. Burguete Cabañas, E. Burte, A. Bush, F. Caballero-Fonseca, D. Caillot, D. Caimmi, M. A. Calderon, P. A. M. Camargos, T. Camuzat, G. Canfora, G. W. Canonica, V. Cardona, K. H. Carlsen, P. Carreiro-Martins, A. M. Carriazo, W. Carr, C. Cartier, T. Casale, G. Castellano, L. Cecchi, A. M. Cepeda Sarabia, N. H. Chavannes, Y. Chen, R. Chiron, T. Chivato, E. Chkhartishvili, A. G. Chuchalin, K. F. Chung, M. M. Ciaravolo, A. Ciceran, C. Cingi, G. Ciprandi, A. C. Carvalho Coehlo, L. Colas, E. Colgan, J. Coll, D. Conforti, J. Correia de Sousa, R. M. Cortés-Grimaldo, F. Corti, E. Costa, M. C. Costa-Dominguez, A. L. Courbis, L. Cox, M. Crescenzo, A. A. Cruz, A. Custovic, W. Czarlewski, S. E. Dahlen, C. Dario, J. da Silva, Y. Dauvilliers, U. Darsow, F. De Blay, G. De Carlo, T. Dedeu, M. de Fátima Emerson, G. De Feo, G. De Vries, B. De Martino, N. de Paula Motta Rubini, D. Deleanu, P. Demoly, J. A. Denburg, P. Devillier, S. Di Capua Ercolano, N. Di Carluccio, A. Didier, D. Dokic, M. G. Dominguez-Silva, H. Douagui, G. Dray, R. Dubakiene, S. R. Durham, G. Du Toit, M. S. Dykewicz, Y. El-Gamal, P. Eklund, E. Eller, R. Emuzyte, J. Farrell, A. Farsi, J. Ferreira de Mello, J. Ferrero, A. Fink-Wagner, A. Fiocchi, W. J. Fokkens, J. A. Fonseca, J. F. Fontaine, S. Forti, J. M. Fuentes-Perez, J. L. Gálvez-Romero, A. Gamkrelidze, J. Garcia-Aymerich, C. Y. García-Cobas, M. H. Garcia-Cruz, B. Gemicioglu, S. Genova, C. George, J. E. Gereda, R. Gerth van Wijk, R. M. Gomez, J. Gómez-Vera, S. González Diaz, M. Gotua, I. Grisle, M. Guidacci, N. A. Guldemond, Z. Gutter, M. A. Guzmán, T. Haahtela, J. Hajjam, L. Hernández, J. O’B Hourihane, Y. R. Huerta-Villalobos, M. Humbert, G. Iaccarino, M. Illario, J. C. Ivancevich, E. J. Jares, E. Jassem, S. L. Johnston, G. Joos, K. S. Jung, M. Jutel, I. Kaidashev, O. Kalayci, A. F. Kalyoncu, J. Karjalainen, P. Kardas, T. Keil, P. K. Keith, M. Khaitov, N. Khaltaev, J. Kleine-Tebbe, L. Klimek, M. L. Kowalski, M. Kuitunen, I. Kull, P. Kuna, M. Kupczyk, V. Kvedariene, E. Krzych-Falta, P. Lacwik, D. Larenas-Linnemann, D. Laune, D. Lauri, J. Lavrut, L. T. T. Le, M. Lessa, G. Levato, J. Li, P. Lieberman, A. Lipiec, B. Lipworth, K. C. Lodrup Carlsen, R. Louis, O. Lourenço, J. A. Luna-Pech, K. Maciej, A. Magnan, B. Mahboub, D. Maier, A. Mair, I. Majer, J. Malva, E. Mandajieva, P. Manning, E. De Manuel Keenoy, G. D. Marshall, M. R. Masjedi, J. F. Maspero, E. Mathieu-Dupas, J. J. Matta Campos, A. L. Matos, M. Maurer, S. Mavale-Manuel, O. Mayora, M. A. Medina-Avalos, E. Melén, E. Melo-Gomes, E. O. Meltzer, E. Menditto, J. Mercier, N. Miculinic, F. Mihaltan, B. Milenkovic, G. Moda, M. D. Mogica-Martinez, Y. Mohammad, I. Momas, S. Montefort, R. Monti, D. Mora Bogado, M. Morais-Almeida, F. F. Morato-Castro, R. Mösges, A. Mota-Pinto, P. Moura Santo, J. Mullol, L. Münter, A. Muraro, R. Murray, R. Naclerio, R. Nadif, M. Nalin, L. Napoli, L. Namazova-Baranova, H. Neffen, V. Niedeberger, K. Nekam, A. Neou, A. Nieto, L. Nogueira-Silva, M. Nogues, E. Novellino, T. D. Nyembue, R. E. O’Hehir, C. Odzhakova, K. Ohta, Y. Okamoto, K. Okubo, G. L. Onorato, M. Ortega Cisneros, S. Ouedraogo, I. Pali-Schöll, S. Palkonen, P. Panzner, N. G. Papadopoulos, H. S. Park, A. Papi, G. Passalacqua, E. Paulino, R. Pawankar, S. Pedersen, J. L. Pépin, A. M. Pereira, M. Persico, O. Pfaar, J. Phillips, R. Picard, B. Pigearias, I. Pin, C. Pitsios, D. Plavec, W. Pohl, T. A. Popov, F. Portejoie, P. Potter, A. C. Pozzi, D. Price, E. P. Prokopakis, R. Puy, B. Pugin, R. E. Pulido Ross, M. Przemecka, K. F. Rabe, F. Raciborski, R. Rajabian-Soderlund, S. Reitsma, I. Ribeirinho, J Rimmer, D. Rivero-Yeverino, J. A. Rizzo, M. C. Rizzo, C. Robalo-Cordeiro, F. Rodenas, X. Rodo, M. Rodriguez Gonzalez, L. Rodriguez-Mañas, C. Rolland, S. Rodrigues Valle, M. Roman Rodriguez, A. Romano, E. Rodriguez-Zagal, G. Rolla, R. E. Roller-Wirnsberger, M. Romano, J. Rosado-Pinto, N. Rosario, M. Rottem, D. Ryan, H. Sagara, J. Salimäki, B. Samolinski, M. Sanchez-Borges, J. Sastre-Dominguez, G. K. Scadding, H. J. Schunemann, N. Scichilone, P. Schmid-Grendelmeier, F. S. Serpa, S. Shamai, A. Sheikh, M. Sierra, F. E. R. Simons, V. Siroux, J. C. Sisul, I. Skrindo, D. Solé, D. Somekh, M. Sondermann, T. Sooronbaev, M. Sova, M. Sorensen, M. Sorlini, O. Spranger, C. Stellato, R. Stelmach, R. Stukas, J. Sunyer, J. Strozek, A. Szylling, J. N. Tebyriçá, M. Thibaudon, T. To, A. Todo-Bom, P. V. Tomazic, S. Toppila-Salmi, U. Trama, M. Triggiani, C. Suppli Ulrik, M. Urrutia-Pereira, R. Valenta, A. Valero, A. Valiulis, E. Valovirta, M. van Eerd, E. van Ganse, M. van Hague, O. Vandenplas, M. T. Ventura, G. Vezzani, T. Vasankari, A. Vatrella, M. T. Verissimo, F. Viart, M. Viegi, D. Vicheva, T. Vontetsianos, M. Wagenmann, S. Walker, D. Wallace, D. Y. Wang, S. Waserman, T. Werfel, M. Westman, M. Wickman, D. M. Williams, S. Williams, N. Wilson, J. Wright, P. Wroczynski, P. Yakovliev, B. P. Yawn, P. K. Yiallouros, A. Yorgancioglu, O. M. Yusuf, H. J. Zar, L. Zhang, N. Zhong, M. E. Zernotti, M. Zidarn, T. Zuberbier, C. Zubrinich, A. Zurkuhlen

**Affiliations:** 1 MACVIA-France, Fondation Partenariale FMC VIA-LR, CHRU Arnaud de Villeneuve, 371 Avenue du Doyen Gaston Giraud, Montpellier, France; 2 INSERM U 1168, VIMA: Ageing and Chronic Diseases Epidemiological and Public Health Approaches, Villejuif, Université Versailles St-Quentin-en-Yvelines, UMR-S 1168, Montigny le Bretonneux, France; 3 Euforea, Brussels, Belgium; 4 KYomed-INNOV, Montpellier, France; 5 iQ4U Consultants Ltd, London, UK; 6 MedScript Ltd, Dundalk, Co Louth Ireland; 7 Laboratoire HP2, Grenoble, INSERM, U1042, Université Grenoble Alpes, Grenoble, France; 8 CHU de Grenoble, Grenoble, France; 9 Conseil Général de l’Economie Ministère de l’Economie, de l’Industrie et du Numérique, Paris, France; 10 UCIBIO, REQUINTE, Faculty of Pharmacy and Competence Center on Active and Healthy Ageing, University of Porto (Porto4Ageing), Porto, Portugal; 11 Center for Health Technology and Services Research- CINTESIS, Faculdade de Medicina, Universidade do Porto, Porto, Portugal; 12 Medida, Lda, Porto, Portugal; 13 Faculty of Health Sciences and CICS – UBI, Health Sciences Research Centre, University of Beira Interior, Covilhã, Portugal; 14 Allergy Center, CUF Descobertas Hospital, Lisbon, Portugal; 15 Imunoalergologia, Centro Hospitalar Universitário de Coimbra and Faculty of Medicine, University of Coimbra, Coimbra, Portugal; 16 ProAR – Nucleo de Excelencia em Asma, Federal University of Bahia, Vitória da Conquista, Brazil; 17 WHO GARD Planning Group, Salvador, Brazil; 18 Allergy Service, University Hospital of Federal University of Santa Catarina (HU-UFSC), Florianópolis, Brazil; 19 Asthma Reference Center, Escola Superior de Ciencias da Santa Casa de Misericordia de Vitoria, Vitória, Esperito Santo Brazil; 20 Division for Health Innovation, Campania Region and Federico II University Hospital Naples (R&D and DISMET), Naples, Italy; 21 CIRFF, Federico II University, Naples, Italy; 22 SOS Allergology and Clinical Immunology, USL Toscana Centro, Prato, Italy; 23 Department of Medical Sciences, Allergy and Clinical Immunology Unit, University of Torino & Mauriziano Hospital, Torino, Italy; 24 Consortium of Pharmacies and Services COSAFER, Salerno, Italy; 25 Unit of Geriatric Immunoallergology, University of Bari Medical School, Bari, Italy; 26 Department of Medicine, Surgery and Dentistry “Scuola Medica Salernitana”, University of Salerno, Salerno, Italy; 27 Center of Excellence in Asthma and Allergy, Hospital Médica Sur, México City, Mexico; 28 Mexico City, Mexico; 29 Puebla, Puebla Mexico; 30 Ciutad Mexico, Mexico; 31 Allergology Department, Centre de l’Asthme et des Allergies Hôpital d’Enfants Armand-Trousseau (APHP), Paris, France; 32 UPMC Univ Paris 06, UMR_S 1136, Institut Pierre Louis d’Epidémiologie et de Santé Publique, Sorbonne Universités, Equipe EPAR, 75013 Paris, France; 33 Epidemiology of Allergic and Respiratory Diseases, Department Institute Pierre Louis of Epidemiology and Public Health, INSERM, UPMC Sorbonne Université, Medical School Saint Antoine, Paris, France; 34 La Rochelle, France; 35 Department of Respiratory Diseases, Montpellier University Hospital, Montpellier, France; 36 UPRES EA220, Pôle des Maladies des Voies Respiratoires, Hôpital Foch, Université Paris-Saclay, Suresnes, France; 37 Reims, France; 38 Division of Internal Medicine, Asthma and Allergy, Barlicki University Hospital, Medical University of Lodz, Lodz, Poland; 39 Department of Prevention of Environmental Hazards and Allergology, Medical University of Warsaw, Warsaw, Poland; 40 Clinic of Children’s Diseases, and Institute of Health Sciences Department of Public Health, Vilnius University Institute of Clinical Medicine, Vilnius, Lithuania; 41 European Academy of Paediatrics (EAP/UEMS-SP), Brussels, Belgium; 42 Clinic of Children’s Diseases, Faculty of Medicine, Vilnius University, Vilnius, Lithuania; 43 Faculty of Medicine, Vilnius University, Vilnius, Lithuania; 44 Woodbrook Medical Centre, Loughborough, UK; 45 Allergy and Respiratory Research Group, Usher Institute of Population Health Sciences and Informatics, University of Edinburgh, Medical School, Edinburgh, UK; 46 Centre of Medical Informatics, Usher Institute of Population Health Sciences and Informatics, The University of Edinburgh, Edinburgh, UK; 47 Allergy Unit, Department of Dermatology, University Hospital of Zurich, Zürich, Switzerland; 48 Center for Rhinology and Allergology, Wiesbaden, Germany; 49 Department of Otorhinolaryngology, Head and Neck Surgery, Universitätsmedizin Mannheim, Medical Faculty Mannheim, Heidelberg University, Mannheim, Germany; 50 Comprehensive Allergy-Centre-Charité, Department of Dermatology and Allergy, Charité - Universitätsmedizin Berlin, Berlin, Germany; 51 Global Allergy and Asthma European Network (GA2LEN), Berlin, Germany; 52 Institute of Medical Statistics, and Computational Biology, Medical Faculty, University of Cologne, Cologne, Germany; 53 CRI-Clinical Research International-Ltd, Hamburg, Germany; 54 Department of Internal Medicine, Medical University of Graz, Graz, Austria; 55 Department of ENT, Medical University of Graz, Graz, Austria; 56 Department of Otorhinolaryngology, Academic Medical Centre, Amsterdam, The Netherlands; 57 Department of Public Health and Primary Care, Leiden University Medical Center, Leiden, The Netherlands; 58 ISGlobAL, Centre for Research in Environmental Epidemiology (CREAL), Barcelona, Spain; 59 IMIM (Hospital del Mar Research Institute), Barcelona, Spain; 60 CIBER Epidemiología y Salud Pública (CIBERESP), Barcelona, Spain; 61 Universitat Pompeu Fabra (UPF), Barcelona, Spain; 62 Allergy Section, Department of Internal Medicine, Hospital Vall ‘dHebron & ARADyAL Research Network, Barcelona, Spain; 63 AQuAS, Barcelona, Spain; 64 EUREGHA, European Regional and Local Health Association, Brussels, Belgium; 65 Rhinology Unit and Smell Clinic, ENT Department, Hospital Clínic, University of Barcelona, Barcelona, Spain; 66 Clinical and Experimental Respiratory Immunoallergy, IDIBAPS, CIBERES, University of Barcelona, Barcelona, Spain; 67 Skin and Allergy Hospital, Helsinki University Hospital, Helsinki, Finland; 68 Association of Finnish Pharmacists, Helsinki, Finland; 69 Department of Lung Diseases and Clinical Immunology, University of Turku, Turku, Finland; 70 Terveystalo Allergy Clinic, Turku, Finland; 71 Department of Pulmonary Diseases, Cerrahpasa Faculty of Medicine, Istanbul University, Istanbul, Turkey; 72 Department of Pulmonary Diseases, Faculty of Medicine, Celal Bayar University, Manisa, Turkey; 73 GARD Executive Committee, Manisa, Turkey; 74 Center for Pediatrics and Child Health, Institute of Human Development, Royal Manchester Children’s Hospital, University of Manchester, Manchester, UK; 75 Allergy Department, 2nd Pediatric Clinic, Athens General Children’s Hospital “P&A Kyriakou”, University of Athens, 11527 Athens, Greece; 76 Department of Otorhinolaryngology, University of Crete School of Medicine, Heraklion, Greece; 77 Woolcock Institute of Medical Research, University of Sydney and Sydney Local Health District, Glebe, NSW Australia; 78 Department of Allergy, Immunology and Respiratory Medicine, Alfred Hospital and Central Clinical School, Monash University, Melbourne, VIC Australia; 79 Department of Immunology, Monash University, Melbourne, VIC Australia; 80 Servicio de Alergia e Immunologia, Clinica Santa Isabel, Buenos Aires, Argentina; 81 Director of Center of Allergy, Immunology and Respiratory Diseases, Santa Fe, Argentina Center for Allergy and Immunology, Santa Fe, Argentina; 82 Universidad Católica de Córdoba, Córdoba, Argentina; 83 Department of Clinical Science and Education, Karolinska Institutet, Södersjukhuset, Stockholm, Sweden; 84 Sachs’ Children and Youth Hospital, Södersjukhuset, Stockholm, Sweden; 85 Institute of Environmental Medicine, Karolinska Institutet, Stockholm, Sweden; 86 Centre for Clinical Research Sörmland, Uppsala University, Eskilstuna, Sweden; 87 Upper Airways Research Laboratory, ENT Department, Ghent University Hospital, Ghent, Belgium; 88 Department of Otorhinolaryngology, Univ Hospitals Leuven, Louvain, Belgium; 89 Academic Medical Center, University of Amsterdam, Amsterdam, The Netherlands; 90 EFA European Federation of Allergy and Airways Diseases Patients’ Associations, Brussels, Belgium; 91 Department of Dermatology and Allergy Centre, Odense University Hospital, Odense Research Center for Anaphylaxis (ORCA), Odense, Denmark; 92 Department of Medicine, Clinical Immunology and Allergy, McMaster University, Hamilton, ON Canada; 93 University Hospital Olomouc, Olomouc, Czech Republic; 94 Peercode BV, Geldermalsen, The Netherlands; 95 Faculty of Medicine, Transylvania University, Brasov, Romania; 96 Division of Allergy/Immunology, University of South Florida, Tampa, USA; 97 Section of Allergy and Immunology, Saint Louis University School of Medicine, Saint Louis, MO USA; 98 Johns Hopkins School of Medicine, Baltimore, MD USA; 99 Department of Otorhinolaryngology, Chiba University Hospital, Chiba, Japan; 100 Nova Southeastern University, Fort Lauderdale, Florida USA

**Keywords:** App, ARIA, Asthma, Care pathways, MASK, mHealth, Rhinitis

## Abstract

mHealth, such as apps running on consumer smart devices is becoming increasingly popular and has the potential to profoundly affect healthcare and health outcomes. However, it may be disruptive and results achieved are not always reaching the goals. Allergic Rhinitis and its Impact on Asthma (ARIA) has evolved from a guideline using the best evidence-based approach to care pathways suited to real-life using mobile technology in allergic rhinitis (AR) and asthma multimorbidity. Patients largely use over-the-counter medications dispensed in pharmacies. Shared decision making centered around the patient and based on self-management should be the norm. Mobile Airways Sentinel networK (MASK), the Phase 3 ARIA initiative, is based on the freely available MASK app (*the Allergy Diary*, Android and iOS platforms). MASK is available in 16 languages and deployed in 23 countries. The present paper provides an overview of the methods used in MASK and the key results obtained to date. These include a novel phenotypic characterization of the patients, confirmation of the impact of allergic rhinitis on work productivity and treatment patterns in real life. Most patients appear to self-medicate, are often non-adherent and do not follow guidelines. Moreover, *the Allergy Diary* is able to distinguish between AR medications. The potential usefulness of MASK will be further explored by POLLAR (Impact of Air Pollution on Asthma and Rhinitis), a new Horizon 2020 project using the *Allergy Diary*.

## Background

Allergic rhinitis (AR) is the most common chronic disease worldwide. Evidence-based guidelines have improved knowledge on rhinitis and made a significant impact on AR management. However, many patients remain inadequately controlled and the costs for society are enormous, in particular due to the major impact of AR on school and work productivity [1, 2]. Unmet needs have identified clearly many gaps. These include (1) suboptimal rhinitis and asthma control due to medical, cultural and social barriers [3, 4], (2) poor understanding of endotypes [5], better characterization of phenotypes and multimorbidities [6], better understanding of gender differences [7], (3) assessment of sentinel networks in care pathways for allergen and pollutants exposures, using symptom variation [8], (4) lack of stratification of patients for optimized care pathways [9] and (5) lack of multidisciplinary teams within integrated care pathways, endorsing innovation in real life clinical trials [8] and encouraging patient empowerment [10, 11].

Mobile health (mHealth) is the use of information and communication technology (ICT) for health services and information transfer [12]. mHealth, including apps running on consumer smart devices (i.e., smartphones and tablets), is becoming increasingly popular and has the potential to profoundly impact on healthcare [13]. Novel app-based collaborative systems can have an important role in gathering information quickly and improving coverage and accessibility of prevention and treatment [14]. Implementing mHealth innovations may also have disruptive consequences [15], so it is important to test applicability in each individual situation [16]. A rapid growth of the health apps market has been seen with an estimated 325,000 health apps available in 2017 for most fields of medicine [17]. Benefits and drawbacks have been estimated for a number of disease [18]. The application of mHealth solutions can support the provision of high quality care to patients with AR or asthma, to the satisfaction of both patients and health care professionals, with a reduction in both health care utilization and costs [19]. Appropriately identifying and representing stakeholders’ interests and viewpoints in evaluations of mHealth is a critical part of ensuring continued progress and innovation [20]. Patient, caregiver and clinician evaluations and recommendations play an important role in the development of asthma mHealth tools to support the provision of asthma management [21]. Smart devices and internet-based applications are already used in rhinitis and asthma and may help to address some unmet needs [22]. However, these new tools need to be tested and evaluated for acceptability, usability and cost-effectiveness.

Allergic Rhinitis and its Impact on Asthma (ARIA) has evolved from an evidence-based guideline using the best evidence based approach [1, 23–25] to care pathways using mobile technology in AR and asthma multimorbidity [26]. ARIA appears to be close to the patient’s needs but real-life data suggest that few patients follow guideline recommendations and that they often self-medicate. Moreover, patients frequently using OTC medications dispensed in pharmacies [27]. Shared decision making (SDM) centered around the patient for self-management should be used more often.

Mobile Airways Sentinel networK (MASK), the Phase 3 ARIA initiative, has been initiated to reduce the global burden of rhinitis and asthma multimorbidity, giving the patient and the health care professional simple tools to better prevent and manage respiratory allergic diseases. More specifically, MASK is focusing on (1) understanding the disease mechanisms and the effects of air pollution in allergic diseases and asthma, (2) better appraising the burden incurred by medical needs and indirect costs, (3) the implementation of multi-sectoral care pathways integrating self-care, air pollution and patient’s literacy, using emerging technologies with real world data using the AIRWAYS ICPs algorithm [28], (4) proposing individualized and predictive medicine in rhinitis and asthma multimorbidity, (5) proposing the basis for a sentinel network at the global level for pollution and allergy and (6) assessing the societal implications of exposure to air pollution and allergens and its consequences on health inequalities globally.

The freely available MASK app (*the Allergy Diary*, Android and iOS) [26] is combined with an inter-operable tablet for physicians and other health care professionals (HCPs [29]), using the same extremely simple colloquial language to manage AR (Visual Analogue Scale: VAS) [30, 31]. It is being combined with data on allergen and pollution exposure (POLLAR).

MASK will be scaled up using the EU EIP on AHA strategy [32]. Phase 4 is starting in 2018 and will focus on “change management”. MASK is supported by several EU grants and is a WHO GARD (Global Alliance against Chronic Respiratory Diseases) research demonstration project (Table 1).

**Table 1:** European Union and World Health Organization links of ARIA and MASK

	Date		WHO	EU
ARIA	1999	Workshop	WHO HQ	
	2003–2013	CC rhinitis and asthma	Montpellier	
	2012–	GARD demonstration project	WHO HQ	
	2004–2010	GA2LEN		FP6
	2011–2015	MeDALL		FP7
MASK	2014–	MACVIA-LR		DG Santé-CNECT
	2014–	GARD demonstration project	WHO HQ	
	2014–	EIP on AHA B3		DG Santé-CNECT
	2015–2016	SPAL		Structural and development funds
	2015–2017	Sunfrail		
	2017–	Twinning		DG Santé-CNECT
	2018–	POLLAR		EIT Health

## Methods

### Users

The *Allergy Diary* is used by people who searched the internet, Apple App store, Google Play or in any other way. The pages of the App are on the Euforea-ARIA website (www.euforea.eu/about-us/aria.html). A few users were clinic patients to whom the app was recommended by their physicians. Users were not requested to complete the diary for a minimum number of days. However, due to anonymization of data, no specific information on the route of access to the app could be gathered [33, 34].

The first question of the App is “I have allergic rhinitis”: Yes/No. We tested the sensitivity and specificity of this question [33]. 93.4% users with a positive answer had nasal symptoms versus 12.1% of users with a negative answer. In the first two versions of the App, allergy was not considered in the user’s questionnaire and AR cannot be differentiated from chronic rhinosinusitis. It is now included in the third version of the App (June 2018) and we will be able to answer more appropriately to this question in the next study. The results of the pilot study were confirmed in over 9000 users.

### Settings

MASK is available in 23 countries and 16 languages. To date (01-09-2018) the app has been used by over 24,000 people.

### Ethics and privacy of data

The Allergy Diary is CE1 registered. The terms of use were translated into all languages and customized by lawyers according to the legislation of each country, allowing the use of the results for research and commercial purposes. The example of the UK terms of use have been provided in a previous paper [33].

#### Geolocation

EU data protection rules have changed since the implementation of the General Data Protection Regulation (Art. 4 para. 1 no. 1 GDPR) [35]. Data anonymization is a method of sanitization for privacy. Anonymization renders personal data “in such a manner that the data subject is not or no longer identifiable” [36]. The European Commission’s Article 29 Working Party (WP29) stated already in 2014 with regards to the Directive 95/46/EC [37] that geolocation information is not only personal data but also to be considered as an identifier itself [38, 39]. Processing personal data by means of an app, like e.g. App Diary, besides Directive 95/46/EC [37] also Directive 2002/58/EC [40] as amended by Directive 2009/136/EC [41] applies.

Geolocation was studied for all people who used the Allergy Diary App from December 2015 to November 2017 and who reported medical outcomes. In contradistinction to noise addition (randomization), k-anonymity [42, 43] is an acceptable method for the anonymization of MASK data (generalization) [44] and results can be used for other databases.

#### Privacy assessment impact

Privacy impact assessments (PIAs), also known as data protection impact assessments (DPIAs) in EU law, is required by GDPR (Article 35 Working Party (WP35). PIA is a systematic process to assess privacy risks to individuals in the collection, use, and disclosure of their personal data. The GDPR introduced PIAs to identify high risks to the privacy rights of individuals when processing their personal data. The assessment shall contain at least:

1. a systematic description of the envisaged processing operations and the purposes of the processing, including, where applicable, the legitimate interest pursued by the controller;
2. an assessment of the necessity and proportionality of the processing operations in relation to the purposes;
3. an assessment of the risks to the rights and freedoms of data subjects and
4. the measures envisaged to address the risks, including safeguards, security measures and mechanisms to ensure the protection of personal data and to demonstrate compliance with this Regulation taking into account the rights and legitimate interests of data subjects and other persons concerned.

When these risks are identified, the GDPR expects that an organization formulates measures to address these risks. Those measures may take the form of technical controls such as encryption or anonymization of data.

The PIA analysis is a self-declarative analysis. In France, the local GDPR representative (*Commission Informatique et Liberté,* CNIL) has provided a software to guide the reflexion around security of personal data and the exposure risks in case of security fails. This software has been used to assess all the risks to be considered through the app uses. The conclusion was that is “negligeable”.

The field is moving very fast. In France, June, 10 2018, the modified law “LIL” (*Loi Informatique et Liberté, 2018*-*493,* https://www.cnil.fr/fr/loi-78-17-du-6-janvier-1978-modifiee) was enacted with a special focus on health-related personal data. Even if the articulation of GDPR and LIL is still unclear, we can anticipate that the app use will remain risk free.

### Allergy Diary

**Fig. 1:**
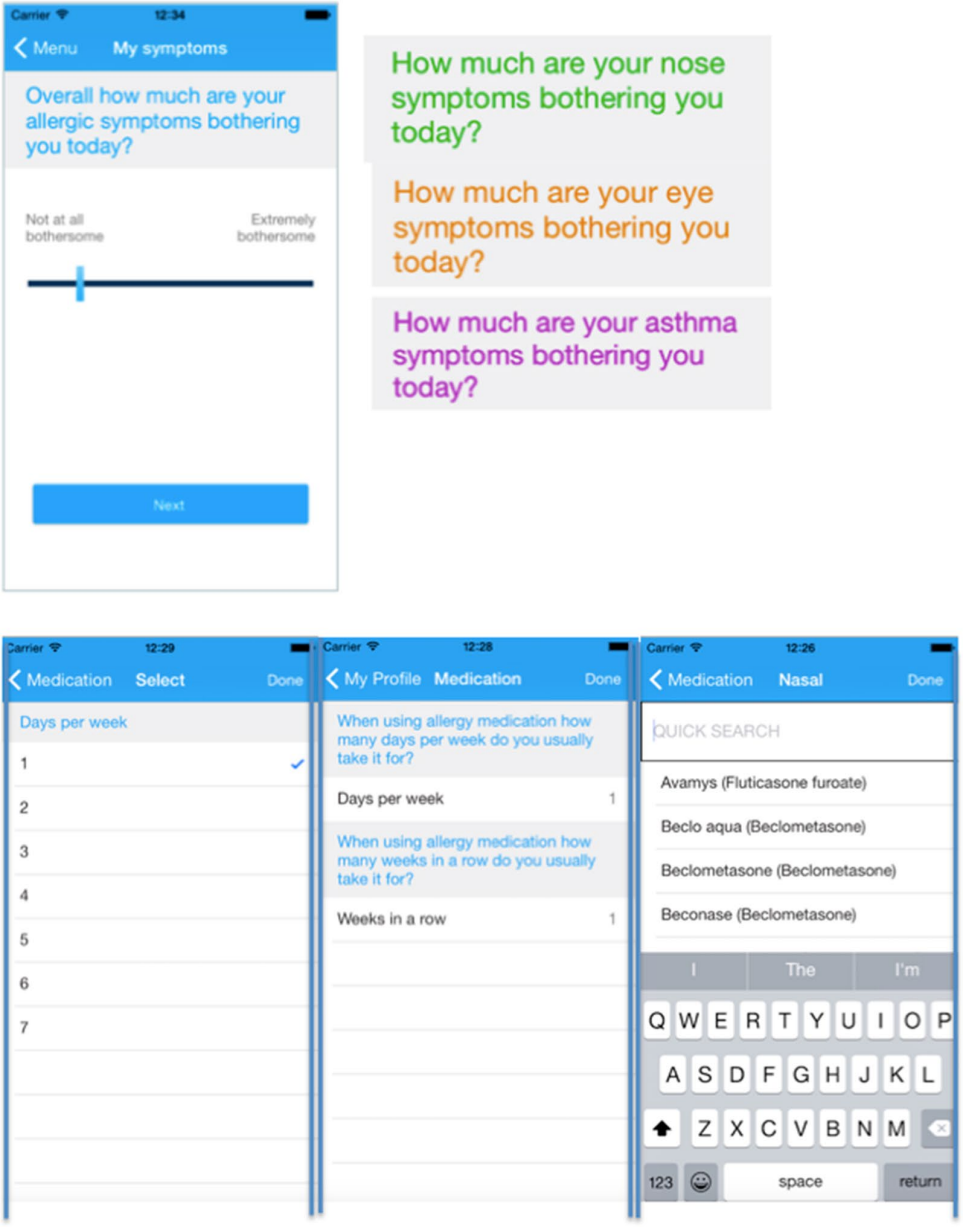
*Allergy Diary* screens relating to Visual Analogue Scale and medications (from Bousquet et al. [26])

**Fig. 2:**
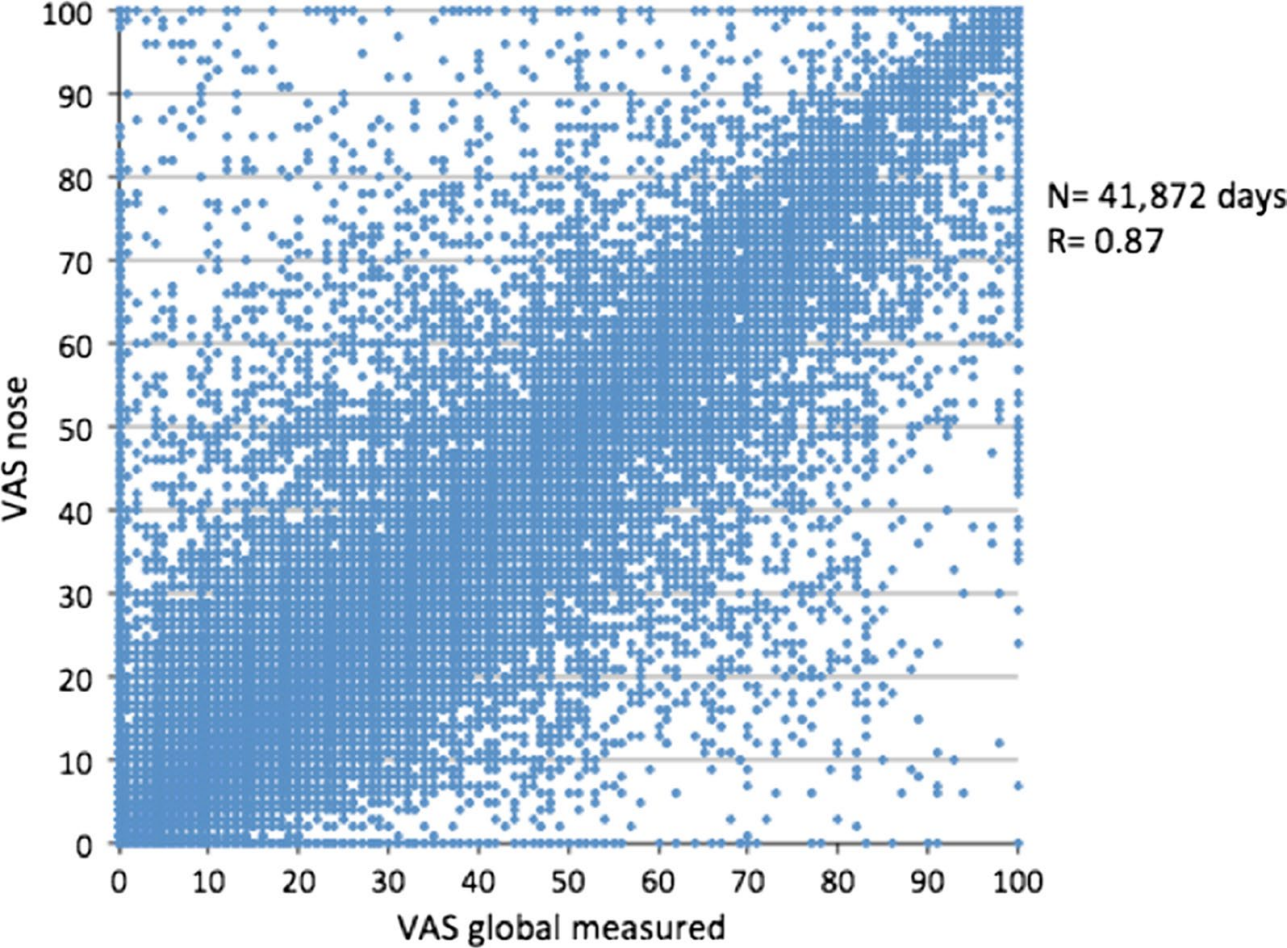
Correlation between Visual Analog Scale (VAS) global measured and nasal symptoms (VAS nose) (unpublished)

The app collects information on AR and asthma symptoms experienced (nasal and ocular) and on disease type (intermittent/persistent) [33] (Table 3). Anonymized and geolocalized users assess daily how symptoms impact their control and AR treatment using the touchscreen functionality on their smart phone to click on five consecutive VAS (i.e. general, nasal and ocular symptoms, asthma and work) (Table 2; Fig. 1). Users input their daily medications using a scroll list that contains all country-specific OTC and prescribed medications available (Fig. 2). The list populated using IMS data and revised by country experts is continuously revised by country experts.

**Table 2:**
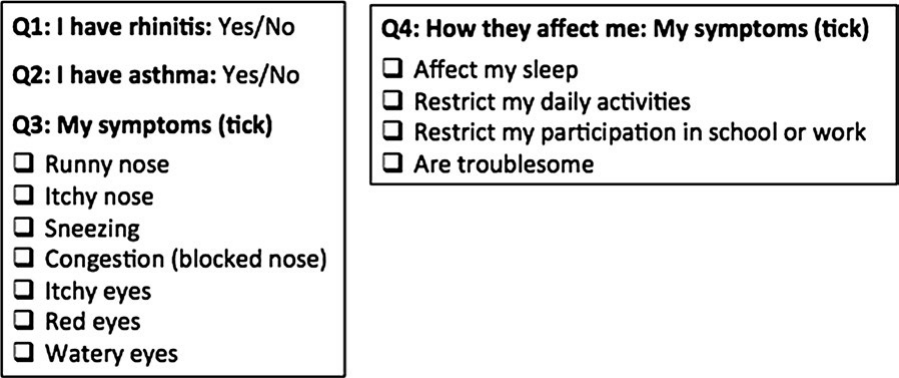
Questions on symptoms and impact of symptoms (from Bousquet et al. [33])

There is a high degree of correlation between these VAS measurements. The example of VAS global measured and VAS nose is presented in Fig. 2.

### Outcomes

Five VAS measurements [VAS-global measured, VAS-nose, VAS-eye, VAS-asthma and VAS-work (Table 4)] and a calculated VAS-global score (VAS-nasal + VAS-ocular divided by 2) were assessed [34]. VAS levels range from zero (not at all bothersome) to 100 (very bothersome). Independency of VAS questions was previously confirmed using the Bland and Altman regression analysis [34, 45].

### Transfer of personal data from the App to a print

**Fig. 3:**
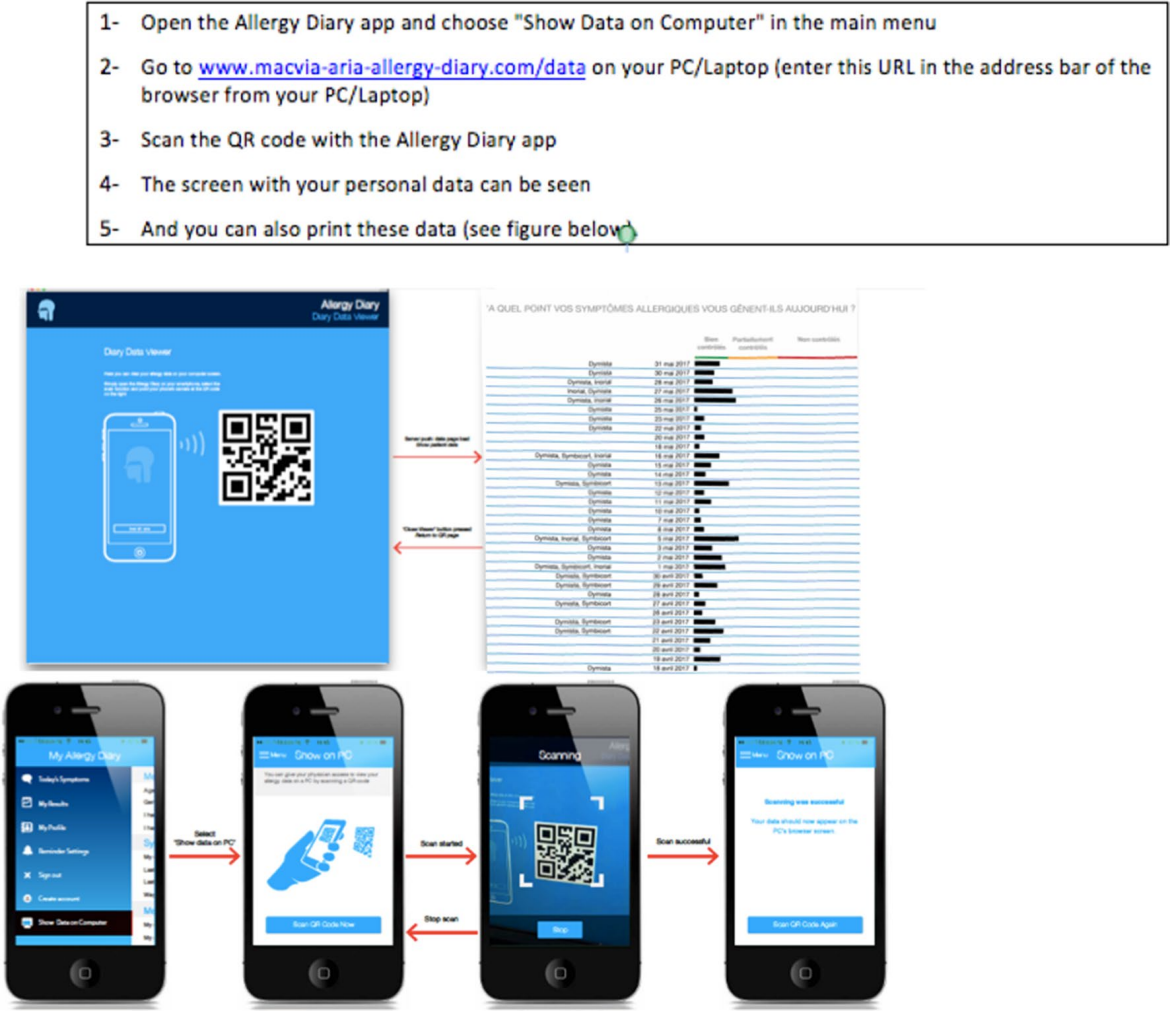
Transfer of patient information on a computer and printed information (from Bousquet et al. [46]

Patients cannot give access to their electronic data to a HCP due to privacy policies. However, they can easily print the daily control of their disease and the medications that they filled in the *Allergy Diary* as follows (Fig. 3).

### Additional questionnaires

MASK also includes EQ-5D (EuroQuol) [46–48], Work Productivity and Activity Impairment Allergic Specific (WPAI-AS) [49] and Control of AR and Asthma Test (CARAT) [50–53]. The Epworth Sleepiness Questionnaire [54, 55] is included (June 2018).

### Medications

A scroll list is available for all OTC and prescribed medications of the 23 countries. The International Nonproprietary Names classification was used for drug nomenclature [56]. 85 INNs and 505 medications were identified (Fig. 1).

### Adherence to treatment

Globally, non-adherence to medications is a major obstacle to the effective delivery of health care. Many mobile phone apps are available to support people to take their medications and to improve medication adherence [57, 58]. However, a recent meta-analysis found that the majority did not have many of the desirable features and were of low quality [57]. However, it is unknown how people use apps, what is considered adherent or non-adherent in terms of app usage, or whether adherence with an app in anyway reflects adherence with medication or control.

In MASK, we did not use adherence questionnaires but first attempted to assess short-term adherence and then to address the long-term issues. [59].

### Digitalized ARIA symptom-medication score

Symptom-medication scores are needed to assess the control of allergic diseases. They are currently being developed for MASK and are being compared with existing ones [60].

### MASK algorithm and clinical decision support system

Clinical decision support systems (CDSS) are software algorithms that advise health care providers on the diagnosis and management of patients based on the interaction of patient data and medical information, such as prescribed drugs. CDSS should be based on the best evidence and algorithms to aid patients and health care professionals to jointly determine the treatment and its step-up or step-down strategy for an optimal disease control.

**Fig. 4:**
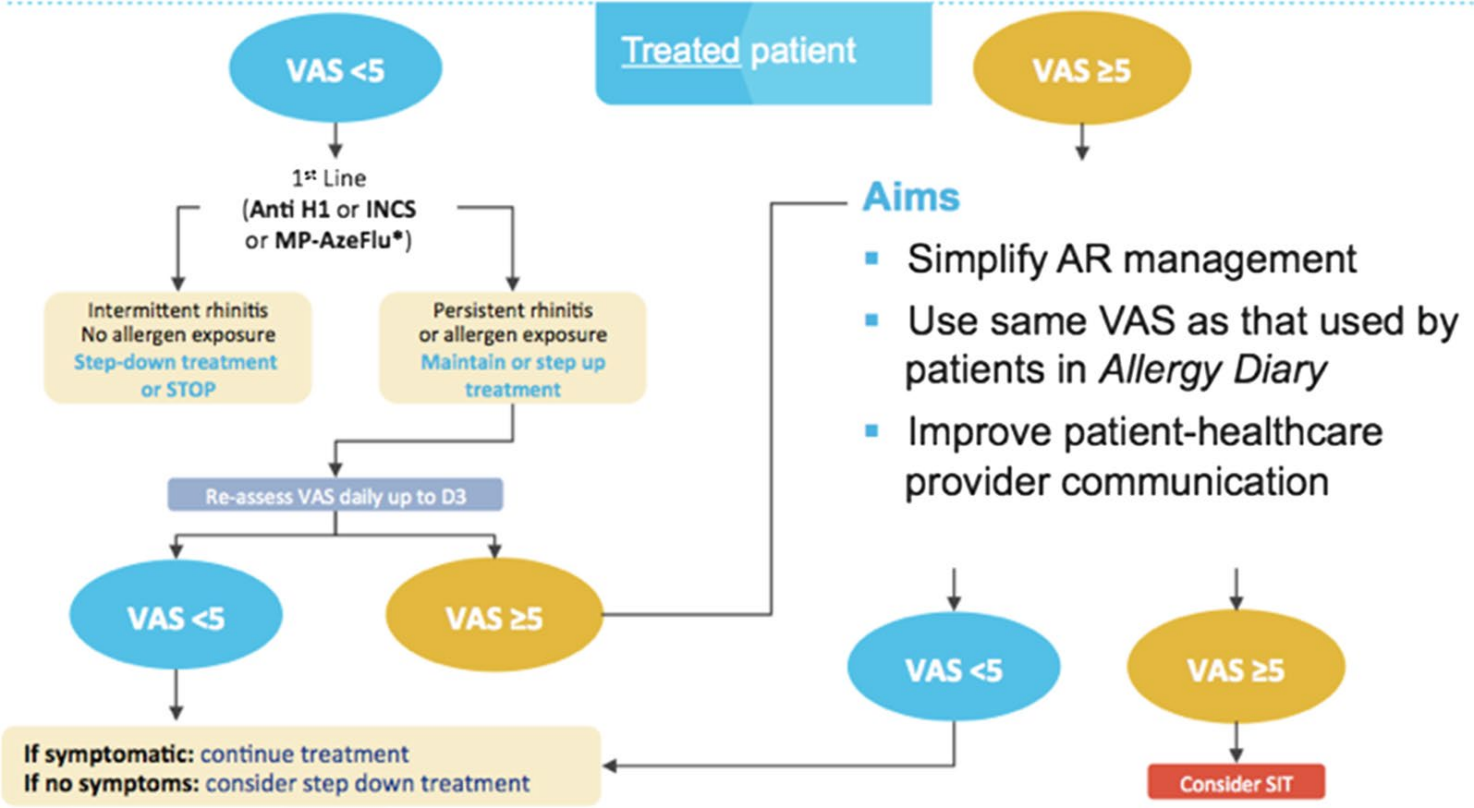
Clinical decision support systems consensus for allergic rhinitis (from Bousquet et al. [28])

**Fig. 5:**
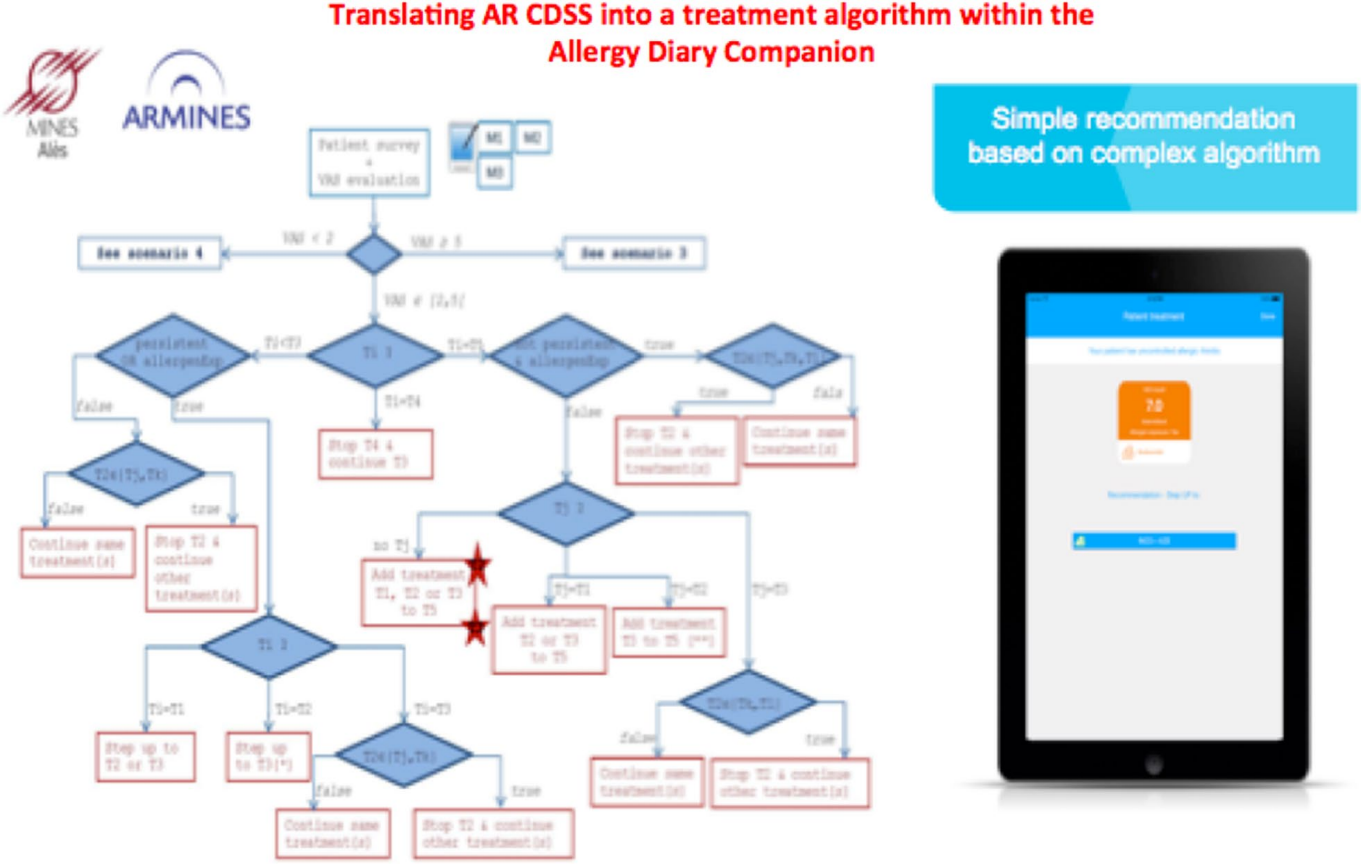
CDSS digitalization (submitted)

The selection of pharmacotherapy for AR patients depends on several factors, including age, prominent symptoms, symptom severity, AR control, patient preferences and cost. Allergen exposure, pollution and resulting symptoms vary, needing treatment adjustment. In AR, The MASK CDSS is incorporated into an interoperable tablet [29] for HCPs (*ARIA Allergy Diary Companion*) [10, 26]. This is based on an algorithm to aid clinicians to select pharmacotherapy for AR patients and to stratify their disease severity [26] (Fig. 4). It uses a simple step-up/step-down individualized approach to AR pharmacotherapy and may hold the potential for optimal control of symptoms, while minimizing side-effects and costs. However, its use varies depending on the availability of medications in the different countries and on resources. The algorithm is now digitalized and available in English (Fig. 5).

## MASK follows the CHRODIS criteria of “Good Practice”

The European Commission is co-funding a large collaborative project named JA-CHRODIS in the context of the 2nd EU Health Programme 2008–2013 [61]. JA-CHRODIS has developed a check-list of 27 items for the evaluation of Good Practices (GP) (http://chrodis.eu/our-work/04-knowledge-platform/). According to the JA-CHRODIS, a Good Practice has been proven to work well and produce good results, and is therefore recommended as a model to be scaled up. The JA-CHRODIS criteria are grouped into nine categories:

- Equity.
- Practice.
- Ethical considerations.
- Evaluation.
- Empowerment and participation.
- Target population.
- Sustainability.
- Governance.
- Scalability

As part of SUNFRAIL, MASK tested the 27 item criteria of CHRODIS and was found to be an example of Good Practice [62].

## Pilot study of mobile phone technology in AR

**Fig. 6:**
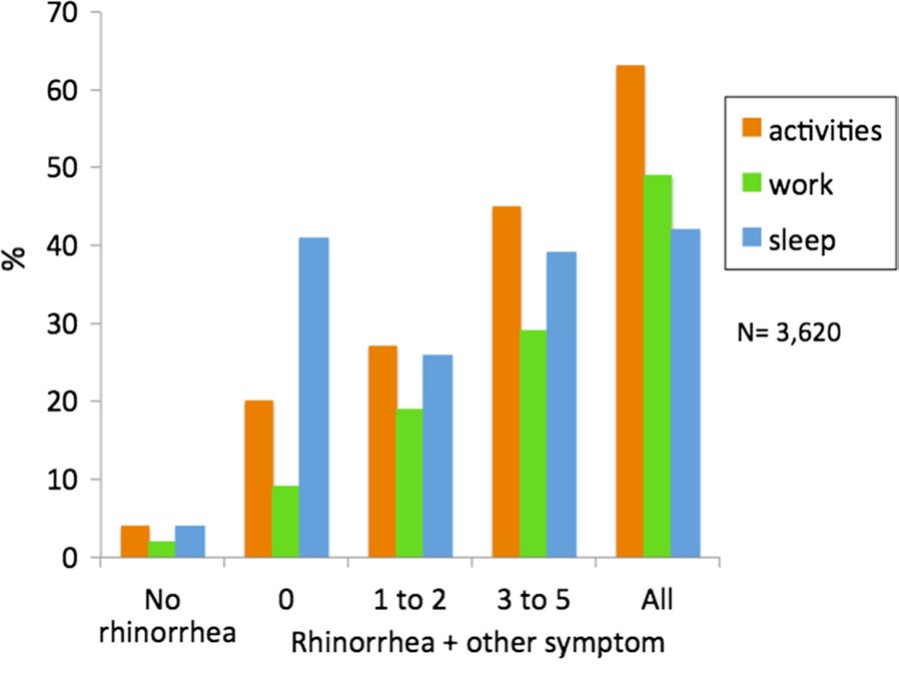
Impact of allergic rhinitis depending on the number of symptoms (from Bousquet et al. [33])

A pilot study in 3260 users found that *Allergy Diary* users were able to properly provide baseline simple phenotypic characteristics. Troublesome symptoms were found mainly in the users with the largest number of symptoms. Around 50% of users with troublesome rhinitis and/or ocular symptoms suffered work impairment. Sleep was impaired by troublesome symptoms and nasal obstruction (Fig. 6). results suggest novel concepts and research questions in AR that may not be identified using classical methods [33].

## Validation of the MASK Visual Analogue Scale on cell phones

VAS included in the *Allergy Diary* was found to be a validated tool to assess control in AR patients following COSMIN guidelines [63] in 1225 users and 14,612 days: internal consistency (Cronbach’s α-coefficient > 0.84 and test–retest > 0.7), reliability (intra-class correlation coefficients), sensitivity and acceptability [64]. In addition, e-VAS had a good reproducibility when users (n = 521) answered the e-VAS twice in less than 3 h.

## Transfer of innovation of AR and asthma multimorbidity in the elderly: Reference Site Twinning (EIP on AHA)

The EIP on AHA includes 74 Reference Sites. The aim of this TWINNING was to transfer innovation from the MASK App to other reference sites. The phenotypic characteristics of rhinitis and asthma multimorbidity in adults and the elderly are compared using validated mHealth tools (i.e. the Allergy Diary and CARAT) in 23 Reference Sites or regions across Europe and Argentina, Australia, Brazil and Mexico [46]. This will improve understanding, assessment of burden, diagnosis and management of rhinitis in the elderly by comparison with an adult population. The pilot study has been completed in Germany and the project is fully operative using two protocols (Table 3).

**Table 3:** Twinning protocols (from Bousquet et al., [65])

	Protocol 1	Protocol 2
	Short version	Long version
Allergy Diary	+	+
Equation 5D	Optional	+
Physician’s questionnaire		+
Ethics committee	Not needed	Needed (obtained in some Reference Sites)
Inform consent	Terms of Reference on App	From with patient’s signature
Recruitment	Any user Persons attending clinic visits can be included	Persons attending clinic visits included with a physician’s diagnosis of allergic disease and allergen sensitization (IgE and/or skin tests)
Physician’s questionnaire		+

## Results

### Work productivity

AR impairs social life, work and school productivity. Indirect costs associated with lost work productivity are the principal contributor to the total AR costs and result mainly from impaired work performance by presenteeism [2]. The severity of AR symptoms was the most consistent disease-related factor associated with impact of AR on work productivity, although ocular symptoms and sleep disturbances may independently affect work productivity. Overall, the pharmacologic treatment of AR showed a beneficial effect on work productivity.

**Fig. 7:**
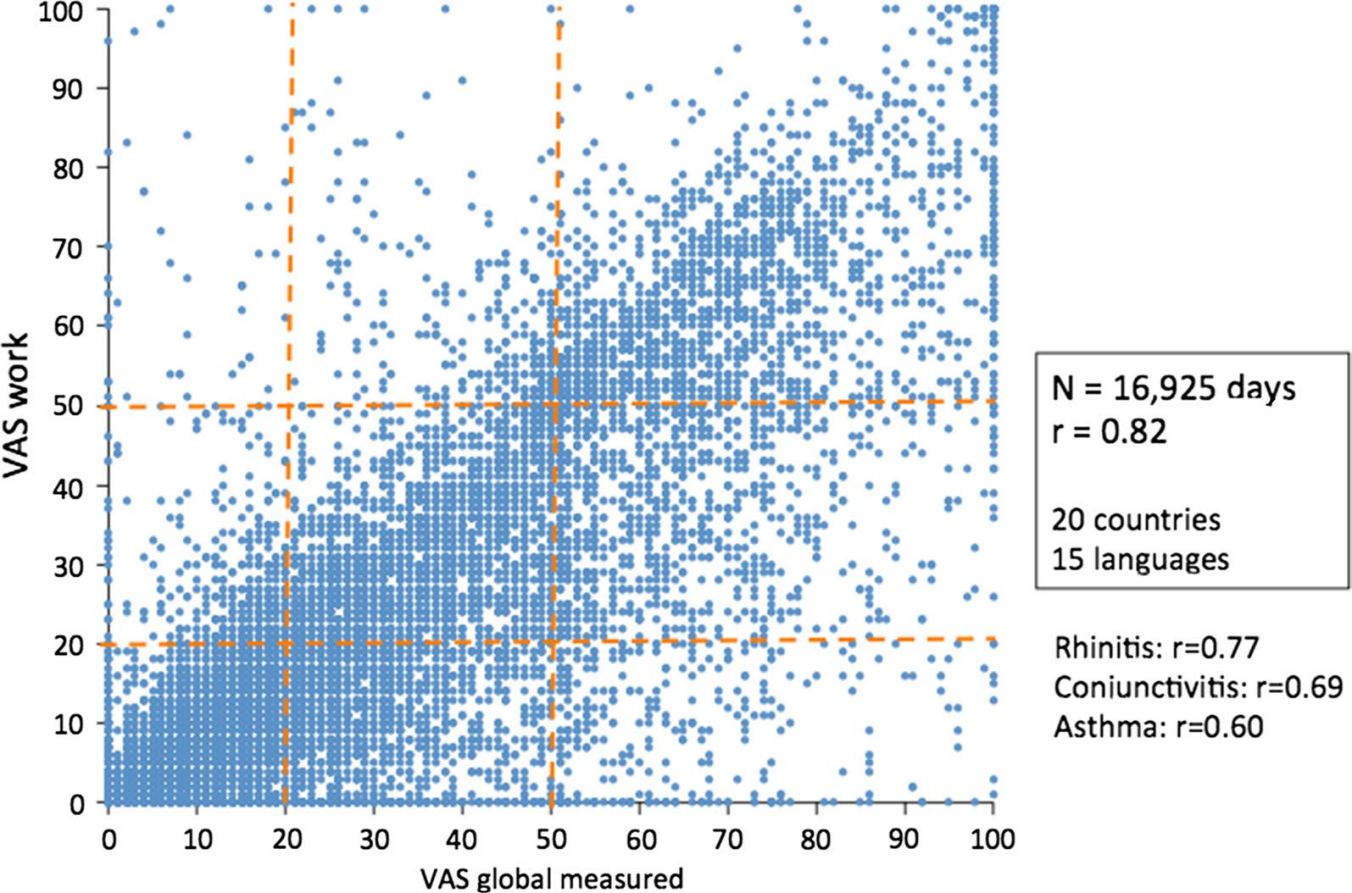
Correlation between VAS work and VAS global measured, nose, eye and asthma (Bousquet unpublished)

A cross-sectional study using *Allergy diary* in 1136 users (5659 days) assessed the impact on work productivity of uncontrolled AR assessed by VAS [34]. In users with uncontrolled rhinitis (VAS global measured ≥ 50), approximately 90% had some work impairment and over 50% had severe work impairment (VAS-work ≥ 50). There was a significant correlation between VAS-global calculated and VAS-work (Rho = 0.83, p < 0.00001, Spearman rank test). The study has been extended to almost 17,000 days and similar results were observed (Fig. 7).

**Fig. 8:**
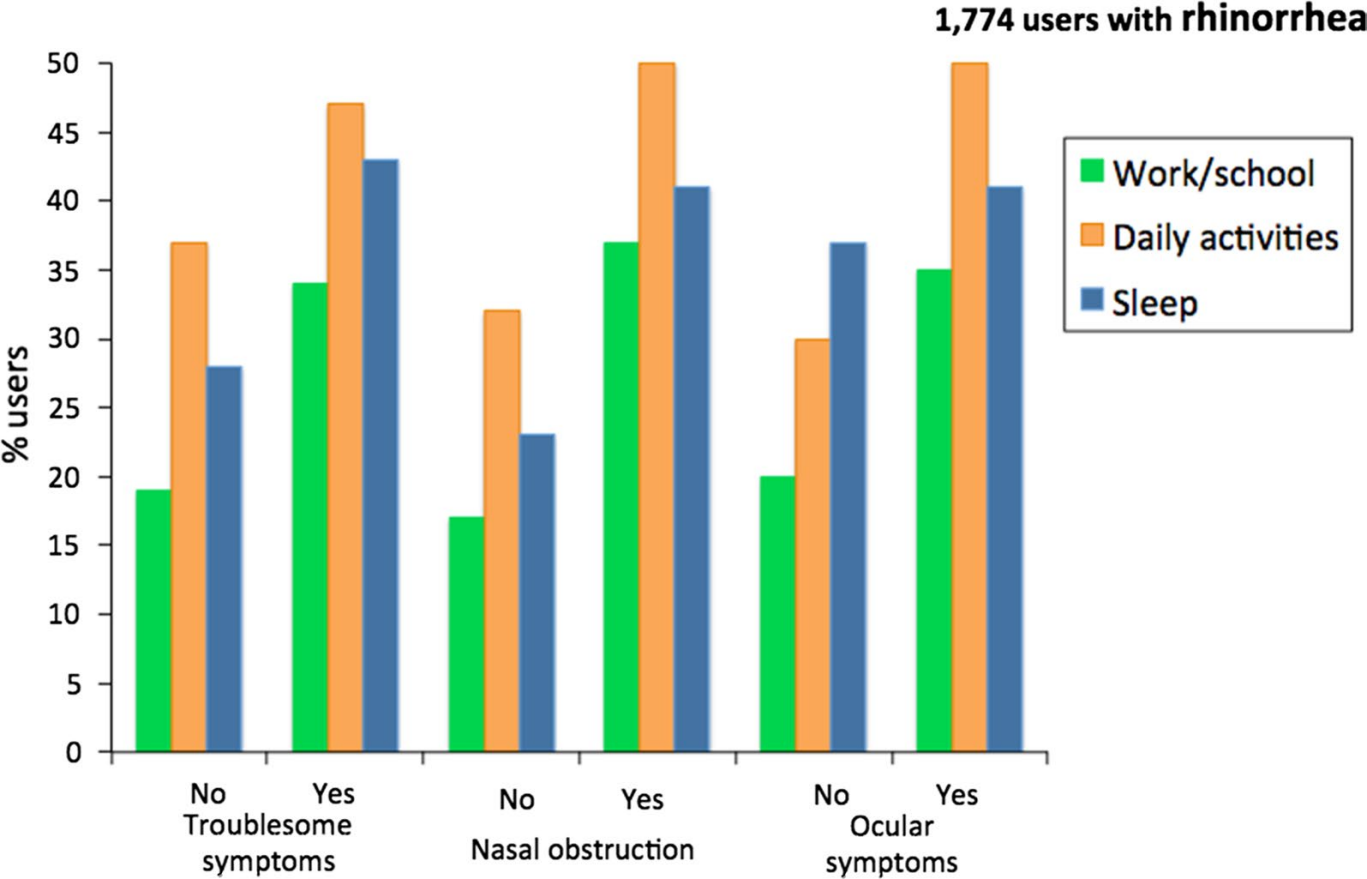
Impact of symptoms on work, school and daily activities (from Bousquet et al. [33])

The baseline study found that bothersome symptoms, nasal obstruction and ocular symptoms were involved in work productivity impact [33] (Fig. 8).

*The Allergy Diary* includes the WPAI:AS in six EU countries. All consecutive users who completed the VAS-work from June 1 to July 31, 2016 were included in the study [66]. A highly significant correlation was found between Questions 4 (impairment of work) and 9 (impairment of activities) in 698 users (Rho = 0.85).

All these studies combine to confirm the impact of uncontrolled AR on work productivity.

### Novel phenotypes of allergic diseases

**Fig. 9:**
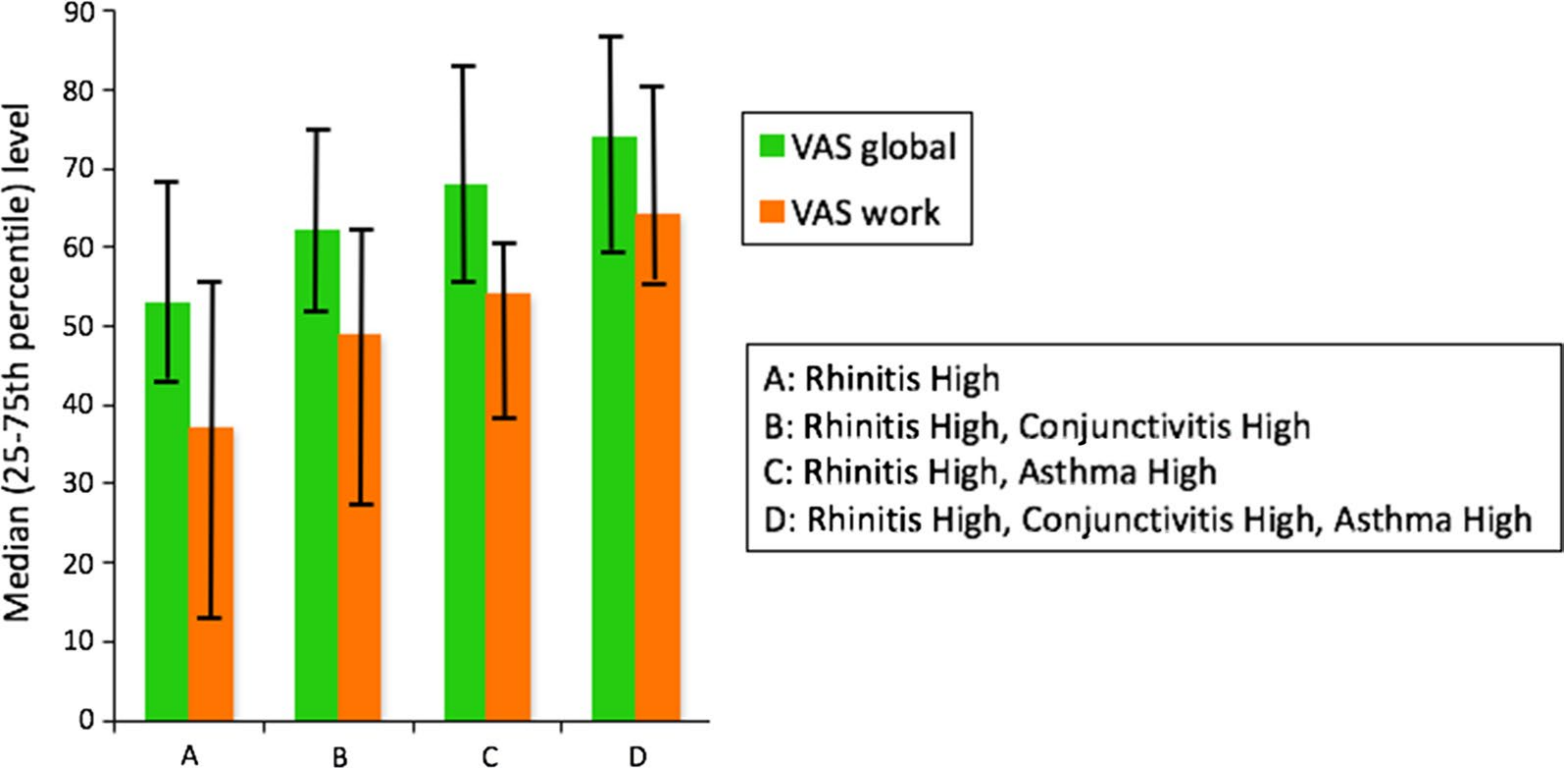
VAS levels in severe rhinitis depending on multimorbidity (from Bousquet et al. [60])

Multimorbidity in allergic airway diseases is well known [6], but no data exist regarding the daily dynamics of symptoms. The *Allergy Diary* assessed the presence and control of daily allergic multimorbidity (asthma, conjunctivitis, rhinitis) and its impact on work productivity in 4025 users and 32,585 days monitored in 19 countries from May 25, 2015 to May 26, 2016. VAS levels < 20/100 were categorized as “Low” burden and VAS levels ≥ 50/100 as “High” burden. VAS global measured levels assessing the global control of the allergic disease were significantly associated with daily allergic multimorbidity. Eight hypothesis-driven patterns were defined based on “Low” and “High” VAS levels. There were < 0.2% days of Rhinitis Low and Asthma High or Conjunctivitis High patterns. There were 5.9% days with a Rhinitis High—Asthma Low pattern. There were 1.7% days with a Rhinitis High—Asthma High—Conjunctivitis Low pattern. A novel Rhinitis High—Asthma High—Conjunctivitis High pattern was identified in 2.9% days and had the greatest impact on uncontrolled VAS global measured and impaired work productivity (Fig. 9). The mobile technology enabled investigation in a novel approach of the intra-individual variability of allergic multimorbidity using days. It identified an unrecognized extreme pattern of uncontrolled multimorbidity [59].

### Treatment of allergic rhinitis using mobile technology with real world data

**Fig. 10:**
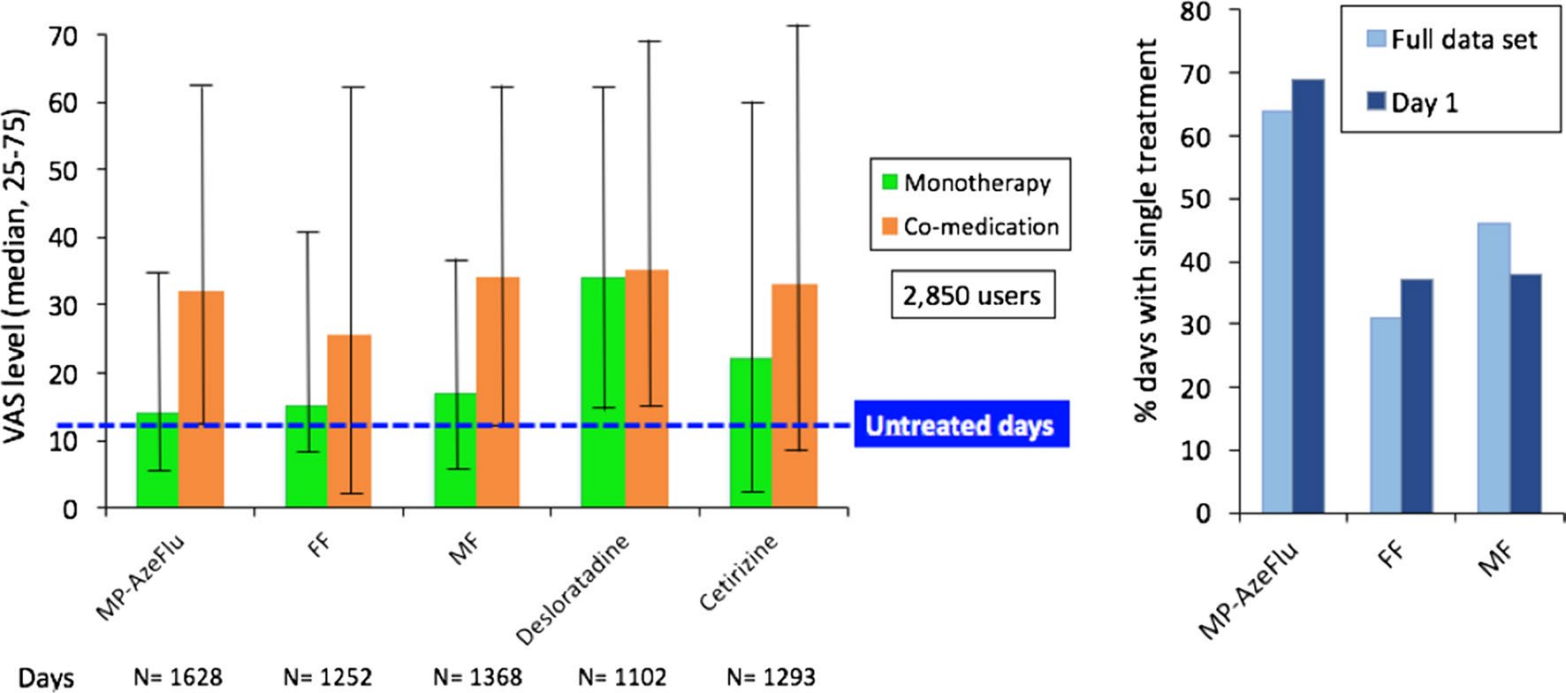
Treatments received in MAS (from Bousquet et al. [59])

Large observational implementation studies are needed to triangulate the findings from randomized control trials (RCTs) as they reflect “real world” everyday practice. We attempted to provide additional and complementary insights into the real-life AR treatment using mobile technology. The *Allergy Diary* was filled in by 2871 users who reported 17,091 days of VAS in 2015 and 2016. Medications were reported for 9634 days. The assessment of days appeared to be more informative than the course of the treatment as, in real life, patients rarely use treatment on a daily basis; rather, they appear to increase treatment use with the loss of symptom control and to stop it when symptoms disappear. The *Allergy Diary* allowed the differentiation between treatments within or between classes (intranasal corticosteroid use containing medications and oral H1-antihistamines). The control of days differed between no (best control), single or multiple treatments (worst control) (Fig. 10). The study confirms the usefulness of the *Allergy Diary* in accessing and assessing everyday use and practice in AR [59].

Adherence to medications was studied in almost 7000 users reporting medications. 1770 users reported over 7 days of VAS between January 1, 2016 and August 31, 2016 and a major lack of adherence to treatment was observed for all medications (Menditto et al., in preparation).

### MASK in the pharmacy

Multidisciplinary integrated care is necessary to reduce the burden of chronic diseases. A significant proportion of patients with AR self-manage their condition and often the pharmacist is the first HCP that a person with nasal symptoms contacts [66, 67]. Pharmacists are trusted in the community and are easily accessible. As such, pharmacists are an important part of the multidisciplinary healthcare team, acting at different steps of rhinitis care pathways.

Pharmacists are important in many areas of intervention in AR:

- Recognizing (identification).
- Risk assessment/stratification.
- OTC treatment.
- Manage refils.
- Patient education.
- Referral to a physician.
- Administration of topical treatment technique and adherence to treatment.

Simple algorithms and tools are essential in the routine implementation of these steps. A first approach was made by ARIA in the pharmacy [68] and is currently being updated using MASK.

## POLLAR (Impact of air POLLution on Asthma and Rhinitis)

AR and asthma are impacted by allergens and air pollution. However, interactions between air pollution, sleep [55, 69] and allergic diseases are insufficiently understood. POLLAR aims at combining emerging technologies [search engine TLR2 (technology readiness level); pollution sampler TLR6, App TLR9] with machine learning to (1) understand effects of air pollution in AR and its impact on sleep, work, asthma, (2) propose novel care pathways integrating pollution and patient’s literacy, (3) study sleep, (4) improve work productivity, (5) propose the basis for a sentinel network at the EU level for pollution and allergy and (6) assess the societal implications of the interaction.

POLLAR will use the freely existing application for AR monitoring (*Allergy Diary*, 14,000 users, TLR8) combined with a new tool allowing queries on allergen and pollen (TLR2) and existing pollution data. Machine learning will be used to assess the relationship between air pollution and AR comparing polluted and non-polluted areas in 6 EU countries. Data generated in 2018 will be confirmed in 2019 and extended by the individual assessment of pollution (Canarin^®^, portable sensor, TLR6) in AR and sleep apnea patients used as a control group having impaired sleep. The geographic information system GIS will map the results.

Google Trends (GT) searches trends of specific queries in Google and reflects the real-life epidemiology of AR. We compared GT terms related to allergy and rhinitis in all European Union countries, Norway and Switzerland from January 1, 2011 to December, 20 2016. An annual and clear seasonality of queries was found in most countries but the terms ‘hay fever’, ‘allergy’ and ‘pollen’—show cultural differences [70]. Using longitudinal data in different countries and multiple terms, we identified an awareness-related spike of searches (December 2016) [70]. In asthma, GTs can identify spikes of mortality as was found in Australia and Kuwait in 2016. However, the usual peaks of asthma during allergen exposure or virus infections cannot be easily monitored [71].

## Global applicability of MASK and POLLAR, and their benefits

Although MASK has been devised to optimize care pathways in rhinitis and asthma multimorbidity, its applicability is far more extensive (Table 4).

**Table 4:** Global applicability of MASK

Applicability	MASK
Clinical practice	Physicians will be able to read the files of the patients in order to
	Optimize treatment for the patient and, in particular, the current or the next pollen season
	Assess and increase the adherence to treatment
	Help for shared decision making
	Prescribe allergen immunotherapy (AIT) more rapidly when the patient is not controlled despite optimal pharmacologic treatment
	Determine the efficacy of AIT in patients
	The Allergy Diary is an essential tool to provide personalized medicine in AR and asthma
Change management	The first results of MASK indicate that many patients are uncontrolled and non-adherent to treatment
	Moreover, they appear to use their medications as needed and not as a regular basis as prescribed
	Change management is needed
Patient empowerment	Better understanding of the symptoms
	Sentinel network linking aerobiology data and control
	Improved adherence
	Self-management
	Patient empowerment
	Messages sent by the App
Clinical trials	For RCTs, it is essential to have clarity on definitions, and relevant tools. The Allergy Diary allows
	To better stratify the patients needing AIT
	To assess the efficacy of AIT during the trial
	To assess the efficacy when AIT is stopped
	Observational studies are of key importance to confirm RCTs and bring new hypotheses for the treatment of AR and asthma
Registration and reimbursement of medicines	Controlled trials designed with a uniform approach will be more easily evaluated by the Health Technology Assessment agencies (such as NICE) for reimbursement. The Allergy Diary uses EQ-5D, a validated measure of utility
	Better understanding of direct and indirect costs
	Controlled trials designed with a uniform approach will help to synchronize data from real-life world regarding clinical effects and safety/tolerability of new drugs (post-marketing pharmacovigilance
Research on mechanisms and genetics	A uniform definition and a collaborative approach to epidemiological, genetic and mechanistic research are important and will be enhanced by the stratification of patients using the *Allergy Diary*
	Different levels of phenotype characterization (granularity) can be applied to assess phenotypic characterization in old age subjects
Epidemiology	In epidemiologic population studies, standardized definitions and tools are fundamental. The Allergy Diary allows novel approaches combining classical cross-sectional and longitudinal studies with real life studies in large populations
Employers	AR and asthma represent a major burden for the employers, and the estimated annual costs in the EU range from 30 to 60 B€. Better control of the disease was shown to reduce costs. The *Allergy Diary* has the potential to improve the control of allergic diseases and to significantly improve work productivity at the EU level
Public health planning	For public health purposes, a perfect patient characterization in real life is needed to identify the prevalence, burden and costs incurred by patients in order to improve quality of care and optimize health care planning and policies
Reduction of inequities	Inequities still exist in the EU for allergic diseases prevalence and burden (not only sex/gender inequities). POLLAR will attempt to understand them and to propose policies and health promotion strategies

For MASK, several steps have been achieved.

## Conclusion

MASK is a novel approach to obtain real-life data concerning rhinitis and asthma multimorbidity and to help patients and physicians for a better SDM. It can be used for multiple purposes in a friendly manner in order to improve the control of allergic diseases in a cost-effective approach.

## Abbreviations

AHA: active and healthy ageing
AIRWAYS ICPs: integrated care pathways for airway diseases
AR: allergic rhinitis
ARIA: Allergic Rhinitis and Its Impact on Asthma
CARAT: Control of Allergic Rhinitis and Asthma Test
CDSS: clinical decision support system
CNIL: Commission Informatique et Liberté
CRD: Chronic Respiratory Disease
DG CONNECT: Directorate General for Communications Networks, Content & Technology
DG Santé: Directorate General for Health and Food Safety
DG: Directorate General
EFA: European Federation of Allergy and Airways Diseases Patients’ Associations
EIP on AHA: European Innovation Partnership on AHA
EIP: European Innovation Partnership
EQ-5D: Euroquol
GARD: WHO Global Alliance against Chronic Respiratory Diseases
GDPR: General Data Protection Regulation
GIS: geographic information system
GP: Good Practice
GT: Google Trends
HCP: health care professional
ICP: integrated care pathway
IMS: Institute of Medical Science
JA-CHRODIS: Joint Action on Chronic Diseases and Promoting Healthy Ageing across the Life Cycle
MACVIA-LR: contre les MAladies Chroniques pour un VIeillissement Actif (Fighting chronic diseases for AHA)
MASK: Mobile Airways Sentinel networK
MeDALL: Mechanisms of the Development of ALLergy (FP7)
mHealth: mobile health
NCD: non-communicable disease
OTC: over the counter
PIA: privacy Impact Assessment
POLLAR: Impact of air POLLution on Asthma and Rhinitis
QOL: quality of life
SCUAD: severe chronic upper airway disease
TRL: technology readiness level
TWINNING: transfer of innovation of mobile technology
VAS: Visual Analogue Scale
WHO: World Health Organization
WPAI-AS: Work Productivity and Activity Questionnaire
