# Correction to: MASK 2017: ARIA digitally-enabled, integrated, person-centred care for rhinitis and asthma multimorbidity using real-world-evidence

**DOI:** 10.1186/s13601-019-0290-7

**Published:** 2019-10-09

**Authors:** J. Bousquet, MASK study group, M. van Hage

**Affiliations:** 1 MACVIA-France, Fondation Partenariale FMC VIA-LR, CHRU Arnaud de Villeneuve, 371 Avenue du Doyen Gaston Giraud, Montpellier, France; 2 INSERM U 1168, VIMA: Ageing and Chronic Diseases Epidemiological and Public Health Approaches, Villejuif, Université Versailles St-Quentin-en-Yvelines, UMR-S 1168, Montigny le Bretonneux, France; 3 Euforea, Brussels, Belgium

## Correction to: Clin Transl Allergy (2018) 8:45 10.1186/s13601-018-0227-6

Following publication of the original article [1], the authors reported that one of the collaborators’ names was spelled incorrectly. In this Correction the incorrect and correct author name are shown. In the author list of this Correction article, only the corresponding author and institutional author are presented.

Originally the collaborator name was published as:

- M. van Hague

The correct collaborator name is:

- M. van Hage
